# Postoperative Deterioration in Health Related Quality of Life as Predictor for Survival in Patients with Glioblastoma: A Prospective Study

**DOI:** 10.1371/journal.pone.0028592

**Published:** 2011-12-09

**Authors:** Asgeir S. Jakola, Sasha Gulati, Clemens Weber, Geirmund Unsgård, Ole Solheim

**Affiliations:** 1 Department of Neurosurgery, St.Olavs University Hospital, Trondheim, Norway; 2 Medical Imaging Lab and Department of Circulation and Medical Imaging, Norwegian University of Science and Technology, Trondheim, Norway; 3 Department of Neuroscience, Norwegian University of Science and Technology, Trondheim, Norway; 4 Department of Laboratory Medicine, Children's and Women's Health, Norwegian University of Science and Technology, Trondheim, Norway; Genentech Inc., United States of America

## Abstract

**Background:**

Studies indicate that acquired deficits negatively affect patients' self-reported health related quality of life (HRQOL) and survival, but the impact of HRQOL deterioration after surgery on survival has not been explored.

**Objective:**

Assess if change in HRQOL after surgery is a predictor for survival in patients with glioblastoma.

**Methods:**

Sixty-one patients with glioblastoma were included. The majority of patients (n = 56, 91.8%) were operated using a neuronavigation system which utilizes 3D preoperative MRI and updated intraoperative 3D ultrasound volumes to guide resection. HRQOL was assessed using EuroQol 5D (EQ-5D), a generic instrument. HRQOL data were collected 1–3 days preoperatively and after 6 weeks. The mean change in EQ-5D index was −0.05 (95% CI −0.15–0.05) 6 weeks after surgery (p = 0.285). There were 30 patients (49.2%) reporting deterioration 6 weeks after surgery. In a Cox multivariate survival analysis we evaluated deterioration in HRQOL after surgery together with established risk factors (age, preoperative condition, radiotherapy, temozolomide and extent of resection).

**Results:**

There were significant independent associations between survival and use of temozolomide (HR 0.30, p = 0.019), radiotherapy (HR 0.26, p = 0.030), and deterioration in HRQOL after surgery (HR 2.02, p = 0.045). Inclusion of surgically acquired deficits in the model did not alter the conclusion.

**Conclusion:**

Early deterioration in HRQOL after surgery is independently and markedly associated with impaired survival in patients with glioblastoma. Deterioration in patient reported HRQOL after surgery is a meaningful outcome in surgical neuro-oncology, as the measure reflects both the burden of symptoms and treatment hazards and is linked to overall survival.

## Introduction

Surgical studies in patients with glioblastoma have focused much on resection grades and maximal safe resection is usually advocated. However, measurements of both extents of resection and safety vary between studies and there are few controlled trials. Due to non-uniform inclusion criteria and assessments of outcomes, direct comparison of results and techniques are difficult, if not impossible [1]. Nevertheless, it seems like resections need to be extensive to improve survival, but the resection grade threshold for a probable clinical benefit remains debated [2]–[4]. Safety is less often assessed and there is no uniform and accepted method for reporting of adverse events in surgical trials [5]. Often clinicians or operating surgeons report clinical outcomes in gross functional scales with a potential of assessment and interest bias.

The combination of this ultimately fatal disease with the delicate balance between potential effect and hazards of surgery makes patients' perioperative HRQOL of particular interest. However, the impact of glioblastoma surgery on patient reported outcomes has not been explored much [6]. We have earlier described possible predictors of HRQOL in patients undergoing glioma surgery. The study clearly demonstrated the devastating effect of acquired deficits on patient reported HRQOL [7]. A recent paper found that surgical acquired deficits may be associated with decreased survival as well [8], but the possible impact of postoperative loss of HRQOL on survival has not been explored.

In the present prospective study we aimed to assess if changes in HRQOL after surgery added any prognostic information to the already established risk factors.

## Materials and Methods

### Ethics statement

All patients included have given their written and informed consent. The Data Inspectorate in Norway approved registration and management of data. The study was approved by the Regional Ethical Committee for Health Region Mid-Norway.

### Methods

Study subjects were recruited from patients aged ≥18 years admitted to our department for scheduled brain tumor surgery, in the period from January 2007 through December 2010. Patients were followed until death or until May 15^th^, 2011. Survival was calculated from the date of surgery. Only patients with histopathological confirmed glioblastoma according to the WHO classification were included in this study. Patients provided written informed consent and filled out the EuroQol 5D (EQ-5D) questionnaire 1–3 days before surgery. A study nurse scored preoperative Karnofsky Performance Status (KPS) on admission. Patient follow-up by a study nurse was scheduled at 6 weeks (median time to follow up: 47 days) after surgery. We decided to use 6 weeks to allow for some recovery from transient surgically induced deficits. In addition, few patients experience significant tumor progression in this time frame. At this time point some patients may have started adjuvant therapy and this could influence the HRQOL, however this is expected to be the same between the groups and therefore unlikely to influence the results. Adverse effects are also quite rare during the initiating phase of adjuvant radiotherapy and/or concomitant temozolomide treatment. Structured interviews were used to assess HRQOL (EQ-5D) using the same questionnaire as preoperatively. The patients were also interviewed about possible complications, acquired and/or worsened deficits (motor, language, vision, unsteadiness and other) and altered mental functions (memory, personality and other) experienced after the procedure. Only patients with complete HRQOL data were included in the analyses. Tumor volumes and resection grades were determined from preoperative and early postoperative MRI volumes using an ellipsoid model (4<$>\raster="rg1"<$>×r^3^/3), as described by others [9]. Gross total resection (GTR) was defined as no visible contrast enhancing tumor tissue on the early (<72 hours) postoperative 1.5 T or 3.0 T MRI scans.

### Study population

Sixty-seven patients with glioblastoma were included from baseline, but 6 (9.0%) patients did not complete the EQ-5D questionnaire after surgery. All patients who did not respond were dead at last follow-up. Three were lost to follow-up as they were already dead or in a terminal condition at 6 weeks, whereas the other three patients who were lost to follow- up lived for a median 30 weeks. The only in-hospital registered complication among these six patients was seizures in one patient who had no seizures preoperatively. Median preoperative HRQOL for these six patients was 0.59 (range 0.27–0.74).

Sixty-one patients had complete EQ-5D forms before and after surgery and were included in the analyses. Clinical characteristics are presented in Table 1. The mean age of included patients was 58 years (range 26–81) and 29 (47.5%) were female. The median preoperative KPS was 80 and 84.7% were functionally independent (KPS 70–100). Thirty eight (62.3%) of the operations were primary and 23 (37.7%) were reoperations.

**Table 1:** Clinical characteristics of the patient population.

Clinical characteristics	No. (%)
Age (mean, range)	58 years (28–81)
Female	29 (47.5)
Preoperative KPS^a^ (median, range)	80 (50–100)
Assumed eloquent^b^	33 (54.1)
Primary operation	38 (62.3)
Tumor volume (median, range)	18.4 cm^3^ (1.1–233.5)
Gross total resection	24 (39.3)
Radiotherapy (now or prior)	56 (91.8)
Temozolomide (now or prior)	46 (75.4)
Acquired neurological deficits	23 (37.7)
Complications	15 (24.6)
Complications leading to reoperation	2 (3.3)

a KPS, Karnofsky Performance Status.

b Eloquence is here defined as grade II and grade III according to the definition by Sawaya et al. [37].

### Surgical procedure

All operations were performed under general anesthesia. The SonoWand® neuronavigation system was available if requested by the surgeon and was used in 56 (91.8%) of the operations. The system utilizes 3D preoperative MRI and updated intraoperative 3D ultrasound volumes to guide resection [10]. In eloquent lesions functional neuronavigation was incorporated utilizing a method described in detail earlier [11], [12]. Functional MRI and diffusion tensor imaging data was incorporated into the system in 19 (31.1%) and 23 (37.7%) of the operations respectively. Sixty (98.4%) of the 61 included patients underwent craniotomy and tumor resection. One patient underwent biopsy only. The median preoperative tumor volume was 18.4 cm^3^ and the median resection grade was 96.3% with GTR achieved in 24 (39.3%) of the patients.

### The EuroQol 5D

EQ-5D is a generic (not developed for any specific patient group) and preference-weighted measure of HRQOL [13]. The questionnaire has been applied to a wide range of health conditions and treatments as well as population based health surveys [14], [15]. There are many different instruments available for researchers interested in assessing HRQOL. We chose to use EQ-5D due to the simplicity of the instrument, to enhance patient perception and perhaps also compliance. Generic instruments such as EQ-5D lack disease specific questions that may be relevant to the patient group (e.g. cognitive functions). Generic instruments may therefore lack sensitivity to measure small benefits or negative consequences of surgery. However, we have earlier demonstrated that EQ-5D shows good correlation to KPS in patients with gliomas and is responsive to new neurological deficit which is highly relevant in this patient group. Also, compared to KPS it offers a more nuanced picture with respect to change after surgery. Since KPS only measures one physical dimension of HRQOL it is insensitive to changes in other dimensions [7]. Another important difference between EQ-5D and KPS is that the latter most often is reported by the physician whereas the former is a patient reported outcome. The EQ-5D has been validated in a Norwegian normal population [16], but so far not in glioma patients. In EQ-5D, five dimensions of HRQOL are scored; mobility, self-care, usual activities, pain/discomfort and anxiety/depression with 3 possible answers to each dimension, i.e ‘no problem’, slight problem' or ‘major problem’. This results in the 243 different possible health states which are transformed into a single index value based on a large survey in the UK population [17]. EQ-5D index value is from −0.594 to 1, where 1 corresponds to perfect health, and 0 to death. Negative values are considered to be worse than death. To provide examples a patient scoring 2, 1, 1, 1, and 2 receives a score of 0.78, while a patient scoring 2, 3, 3, 2 and 2 receives a score of 0.08. A visual analogue scale where patients rate their current health state on a line ranging from 0–100 (worst to best imaginable health) forms the second part of the EuroQol questionnaire. In this study only the index value was assessed.

### Statistics

All analyses were done with the PASW statistics, version 18.0. Statistical significance level was set to P<0.05. Q-Q plots were used to test for normal distribution of data. Central tendencies are presented as means if data is normally distributed and as medians when skewed. When analyzing changes in EQ-5D (e.g. before and after surgery) paired sample t-test was used. For comparison of groups with skewed distribution we utilized Mann-Whitney U test. For binominal data we used Pearson's chi square test.

In the Cox multivariate survival analysis the variables were chosen on the basis of current evidence. The most consistent factors affecting survival in patients with glioblastoma are age [18] and preoperative functional status, usually evaluated with Karnofsky Performance Status (KPS) [19], [20]. High quality evidence for the efficacy for adjuvant treatment with radiotherapy and temozolomide in selected patients is now available [21], [22]. There is also growing evidence suggesting that achieving gross total resection improves survival [2], [23]. We performed univariate analyses for each risk factor and included all in the multivariate model. The Cox multivariate model included the following variables: Age (linear), extent of resection (linear), radiation (yes, no), temozolomide (yes, no), preoperative Karnofsky (linear) and deterioration in patient reported HRQOL (yes/no). We are aware that use of linear data is preferable for statistical reasons (no loss of information), but dichotomizing variables makes clinical interpretation easier, especially when scores consist of several summarized variables, making the immediate interpretation of a number less intuitive. For radiation and temozolomide “yes” indicates that the treatment has been provided at any time during the course of the disease.

## Results

### HRQOL evaluated with EQ-5D

The mean preoperative EQ-5D index was 0.67 compared to 0.62 postoperatively. The mean decline of −0.05 (95% CI −0.15–0.05) is a non-significant change (p = 0.285). There was a wide range in the difference (−0.96 to 0.87) after surgery. There were 30 patients (49.2%) who reported a deterioration 6 weeks after surgery while 9 (14.8%) were unchanged and 22 (36.1%) reported improved HRQOL. Treatment and outcome characteristics comparing the patients with deterioration in HRQOL with the others are presented in Table 2. Patients who reported deterioration in HRQOL had EQ-5D index 0.41 postoperatively as compared to 0.81 in their counterparts (p<0.001). The group of patients who experienced a deterioration in HRQOL after surgery (n = 30) more often had acquired deficits (p = 0.017). There was also a trend for better HRQOL preoperatively (p = 0.051), although not statistically significant.

**Table 2:** Comparisons of treatment related factors and outcome among patients experiencing deterioration in HRQOL after surgery with patients with equal or better HRQOL after surgery.^a^

	Deterioration in HRQOL (n = 30)	Equal or improved HRQOL (n = 31)	P-value
Primary operation^b^	17 (56.6%)	21 (67.7%)	0.375
KPS (median) preop^c^	80	90	0.586
Tumor volume (median)^c^	24.1 cm^3^	15.9 cm^3^	0.322
Extent of resection (median)^c^	95.1%	96.5%	0.715
Gross total resection^b^	11 (36.7%)	13 (41.2%)	0.532
Complication^b^	8 (26.7%)	7 (22.6%)	0.401
New/worse deficit^b^	16 (53.3%)	7 (22.6%)	0.017
EQ-5D index (mean) preop^d^	0.75	0.59	0.051
EQ-5D index (mean) postop^d^	0.41	0.81	<0.001
Deaths in month 0–6^b^	11 (36.7%)	3 (9.7%)	0.012
Deaths in month 7–12^b^	6 (20.0%)	5 (16.1%)	0.694
Deaths >12 months^b^	6 (20.0%)	8 (25.8%)	0.590
Total deaths in follow up^b^	23 (76.7%)	16 (51.6%)	0.042

a HRQOL, health related quality of life; KPS, Karnofsky Performance Status; p<0.05 is considered significant.

b Pearson chi-square.

c Mann-Whitney U test.

d Independent sample t-test.

### Survival

**Figure 1:**
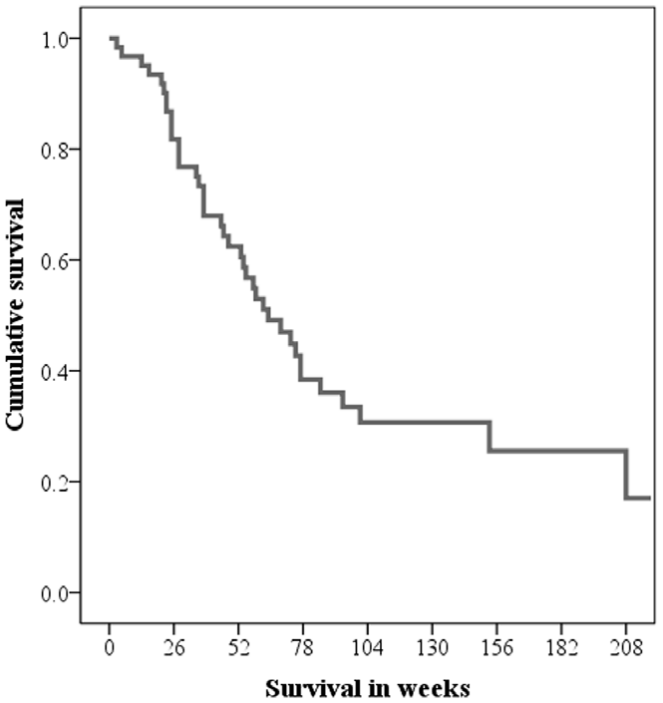
Overall survival in the cohort (n = 61) presented in a survival plot.

At the end of follow up 22 patients (36%) were still alive. Median survival was 64 weeks (95% CI 44–84) and a survival curve is presented in Figure 1.

**Figure 2:**
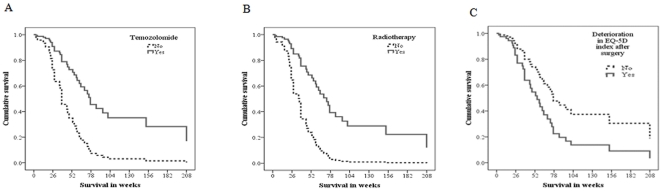
Survival curves for the independent predictors presented in **Table 3**.

In a Cox multivariate survival analysis we evaluated the impact of the established risk factors together with deterioration in HRQOL. The results are presented in Table 3 and Figure 2A, 2B and 2C. There were independent associations between survival and use of temozolomide (HR 0.30, p = 0.019, Figure 2A), radiation therapy (HR 0.26, p = 0.030, Figure 2B), and deterioration in HRQOL after surgery (HR 2.02, p = 0.045, Figure 2C). Patients with deterioration in HRQOL more often died during the first six months following surgery (TYable 2, p = 0.017). Preoperative KPS or surgical extent of resection did not reach statistical significance. Using KPS as a dichotomous variable (KPS≥70) or categorical values for extent of resection (gross total, subtotal and biopsy) did not change the conclusion. Inclusion of surgically acquired deficits in the model did not alter the conclusion either, and actually strengthened the association between deterioration in HRQOL after surgery with overall survival (HR 2.4, p = 0.022). Since requested in the review process, primary and redo operations were analyzed separately. Ad-hoc testing verified that temozolomide and radiation therapy were statistically significant predictors (p<0.05) when the 38 primary operations were analyzed separately. Deterioration in HRQOL did not reach statistical significance (HR 2.9, p = 0.05). No statistically significant predictor was found when analyzing the 23 reoperations separately.

**Table 3:** Cox multivariate regression.^a^

	HR univariate	P-value	HRMultivariate	P-value	95% CI for multivariate HR	
					Lower	Upper
Age	1.04	0.023	1.00	0.990	0.97	1.03
EOR	0.99	0.176	0.99	0.403	0.97	1.00
Radiotherapy	0.12	<0.001	0.26	0.030	0.08	0.88
Temozolomide	0.20	<0.001	0.30	0.019	0.11	0.82
KPS preoperative	0.98	0.083	0.99	0.325	0.96	1.01
HRQOL deterioration	2.11	0.022	2.02	0.045	1.02	4.00

All variables included in the model are presented both for univariate and multivariate analyses. Radiotherapy, use of temozolomide and deterioration in quality of life 6 weeks after surgery were independently associated with overall survival.

a EOR, extent of resection; KPS, Karnofsky Performance Status; HRQOL, health related quality of life; HR, hazard ratio; CI, confidence interval; p<0.05 is considered significant.

## Discussion

In this prospective follow-up study of 61 glioblastoma patients we found that deterioration in HRQOL early after surgery seems to be an independent negative prognostic factor for survival. Deterioration in HRQOL occurs in about half of the patients despite the use of modern image guided surgery. The effect of deteriorating HRQOL was independent of the established risk factors, such as age, extent of resection, preoperative functional status (KPS), and adjuvant treatment. The difference in survival appears to be due to a difference in early mortality. A decline in HRQOL in the early postoperative phase may be suggestive a rapidly growing lesion or perhaps negative effects from surgery. It has been reported that acquired deficits can be associated with both suboptimal adjuvant therapy [5] and reduced survival [8]. Still, we found that the negative impact of lost HRQOL remained significant after adjustment for reported acquired neurologic deficits. Our findings indicate that evaluation of the patients' perception of own health may be of high prognostic value. If so, this may allow for new and interesting outcome measures in glioblastoma surgery that reflect the biology of the disease, the tolls and the benefits from surgery, while maintaining the relevance for overall survival. HRQOL measures allow for comparisons across studies and techniques while avoiding the potential bias associated with surgeons' evaluation of own results.

Overall survival is considered the gold standard when evaluating treatment of glioblastoma and its role is indisputable. However as survival benefits from surgery can be modest, survival as study end-point may require multicentre inclusion and years of recruitment to avoid a statistical power shortage, as experienced in the 5-ALA study [9]. Further, this measure can be quite unspecific in a surgical setting as it reflects the results from non-surgical interventions as well. Progression free survival (PFS) may be used instead as in the 5-ALA-study [9], but the definitions vary and interpretation is problematic [24]. Pseudoprogression occurs in approximately 20% and this makes a pure imaging based outcome unreliable [25]. There may be contrast enhancement due to the treatment itself which can be impossible to distinguish from recurrent disease [24]. Another problem is pseudoresponses, seen with antiangiogenic agents where the disappearance of contrast enhancement is not necessarily related a clinical response [24], [26]. However, the dynamics of tumor progression, the speed of growth, and patterns of growth may be of prognostic importance if a reliable measure becomes available.

Extensive resections are advocated by numerous studies due to the association with improved survival. The association seems logical, but it is difficult to differentiate the efficacy of treatments from treatment selection as most studies are neither randomized, controlled nor prospective [4], [23], [27]. As mentioned earlier, differences in patient selection are obstacles for meaningful comparisons between institutions and techniques. Lastly, with the exception of the 5-ALA study [9] most studies are not even designed to evaluate the efficacy of surgical treatment. The present study does not indicate that extensive resection negatively affects HRQOL in itself, but it indicates that there is serious potential for harm in surgical treatment of glioblastomas. We believe careful therapeutic considerations should be made in cases where safe gross total resection seems unlikely as the risk might outweigh the benefit.

These common end-points all have drawbacks which can be problematic for meaningful clinical interpretation. Since the prognosis with respect to survival remains unfavorable despite maximal therapeutic efforts, measuring patients' quality of survival is an important supplement [6]. We believe HRQOL adds useful information both for clinical use and research. Met with the individual patients, neurosurgeons should take into account the potential hazards of surgery on patients' HRQOL and carefully weigh this up against the likelihood of a survival benefit. Perhaps the patients' subjective HRQOL reflects the dynamics of their disease of prognostic importance, although difficult to quantify even in serial MRI scans. HRQOL reflects both the burden of treatment and the severity of the disease and together with the association to overall we believe that deterioration in HRQOL, or deterioration free survival after surgery, can be a meaningful endpoint in surgical trials in neuro-oncology.

In demonstrating prognostic potential of self reported HRQOL we are in line with earlier studies [28]–[31]. However, we are not aware of any other study assessing the prognostic effect of HRQOL where baseline scores are collected preoperatively. Other neuro-oncological studies evaluating HRQOL and survival are usually in the setting of medical clinical trials using initiation of chemotherapy or radiotherapy as baseline [29]–[32]. This neglects the potential effect and hazards of surgery which undoubtedly is the most invasive form of treatment in patients with glioblastoma.

Patients may perceive their health and HRQOL differently with regards to sex, tumor location and histopathology [6], [7]. Therefore it is difficult to find an optimal cut-off-value with clinical significance, and searching for a so called “best cut-off” may be somewhat dubious and increase the risk for false positive findings [33]. Utilizing changes instead of absolute values seems clinically more useful in individual patients. This approach takes individual differences into consideration as patients are their own controls. This approach may reduce the problems mentioned above. However, interpreting changes in HRQOL is not necessarily straightforward. Changes should be evaluated as clinically meaningful rather than simply statistically significant. This can be achieved by anchoring HRQOL to therapy, changes with disease progression or life events [34].

EQ-5D, a generic HRQOL measure, shows good correlation with traditional outcome measures [7], and in this study it also demonstrates an association with hard clinical end-points. Thus it is seemingly a valuable tool in assessing HRQOL in patients with glioblastoma. Despite potential shortcomings of generic instruments, we are convinced that patient related outcomes with a validated questionnaire are interesting, valuable, and perhaps less biased adjuncts to traditional physician rated outcome measures. The use of EQ-5D for the entire glioblastoma patient population should be subject of further studies i.e. defining minimal important change or measuring HRQOL at multiple time points to better understand the HRQOL throughout the course of the disease. However, we would insist on using a preoperative evaluation as baseline to avoid loss of important information.

The relative high number of complications and acquired deficits in our patients are most likely explained by the assessment method used. All adverse events were patient reported, including uncommon outcome parameters used in the neurosurgical literature, namely memory difficulties, unsteadiness and personality changes. When using a more common method of assessment we have reported complications in 21% and deteriorated functional outcome in 13% in a consecutive, unselected series in patients with high grade gliomas [1]. Comparing adverse events between studies is difficult due to different inclusion criteria and the lack of a standardized way of reporting [5]. With this in mind we believe these findings are comparable to a large study where 34% of patients experienced perioperative complications and 9.9% displayed worsened neurological status within 3 weeks after primary craniotomy for malignant glioma [35]. For the future we would encourage researches to use one standard way of reporting since this would facilitate meaningful comparisons, i.e. using the system for neurosurgical patients recently described [36].

Our study has several limitations. The patients included represent an unsystematic selection that may not be representative for the entire population of patients with glioblastoma. We believe the lost-to follow-up rate of 9% is low. How these lost-to-follow-ups would have affected the results remains speculative, but as three were dead or in a terminal condition, it is reasonable to believe their HRQOL had deteriorated as well and further strengthened the association. Adjuvant treatment (yes/no) was included in the Cox regression model in spite of the risk of survivorship effect overestimating the actual effect of the intervention. A case-mix with 37.7% reoperated patients where most had already received adjuvant treatment could possibly lead to underestimation of the effect of adjuvant treatment. Although the effect of lost HRQOL seems independent of given adjuvant treatment, details of treatment protocols were not studied. We therefore advise to interpret the effects of adjuvant therapy in this study with some caution. Results from the ad-hoc analyses for primary operations and reoperations separately, as requested in the review process, may likely be due to type II errors and should not alter the interpretation of the study. They suggest that the findings in this study may be more representative for primary operations than for reoperations, but this finding needs to be verified in a larger study. Finally, the statistical method used in creating a dichotomous variable (worse HRQOL: yes/no) from a single variable is associated with an increase in false positive findings [33]. However the cut-off chosen is not created on the basis of finding the “optimal” cut-off, but out of logic and what we thought would be of clinical relevance. Another important statistical culprit is the floor-ceiling effect since patients in a good preoperative condition can only become worse and vice versa.

### Conclusion

Balancing risks with potential survival benefit and clinical improvement is the key in surgical treatment of patients with glioblastoma. Resection grades, overall survival, and PFS are much used outcome parameters in surgical research, but they offer no information on quality of survival. In this study we have demonstrated that early deterioration in HRQOL after surgery is independently and markedly associated with impaired survival. Deterioration in patient reported HRQOL after surgery is a meaningful outcome in surgical neuro-oncology as HRQOL reflects the burden of symptoms, the treatment hazards and is linked to survival.
